# RCAS-RNAi: A loss-of-function method for the developing chick retina

**DOI:** 10.1186/1471-213X-6-2

**Published:** 2006-01-22

**Authors:** Sanjiv Harpavat, Constance L Cepko

**Affiliations:** 1 Department of Genetics and Howard Hughes Medical Institute, Harvard Medical School, Boston, USA

## Abstract

**Background:**

The embryonic chick provides an excellent model system for studies of development. However, it has lacked an efficient loss-of-function method for studies of gene function.

**Results:**

We show that avian retroviruses can deliver hairpins mediating RNA interference to the developing chick eye. These viruses 'knock down' specific genes in infected areas of the retina. The knock down persists as the retina matures and can be detected using in situ hybridization. Furthermore, the amount of retinal tissue affected can be controlled by manipulating the degree of infection.

**Conclusion:**

This technique provides a rapid and efficient loss-of-function option for studies in the developing chick retina.

## Background

Chick embryos have long been a favorite of developmental biologists, in large part because they are amenable to in ovo experimentation. However, until recently the chick has lacked a robust lost-of-function technique for studies of gene function. Chick gene "knock outs" are impractical because ES cells are elusive, flocks are unwieldy, and generation times are long [1]. Chick loss-of-function studies, as a result, have resorted to indirect methods such as dominant negative alleles (e.g. transcription factors fused to the engrailed repressor) [2]. Other approaches have included the use of chemical inhibitors to reduce gene function, such as cyclopamine for hedgehog action and SU5402 for fibroblast growth factor signaling [3,4].

More recently, RNA interference (RNAi) techniques have been used successfully in various chick tissues [5-8]. Chick embryos electroporated with small interfering RNAs (siRNAs) show knock down of the targeted gene. However, electroporation techniques are transient, because the effects disappear once cells lose the introduced nucleic acid. Another more stable delivery method uses the Replication Competent Avian Splice (RCAS) retroviruses to introduce hairpins into tissues. Our group and others have used such vectors to reduce gene function in a variety of tissues, including developing craniofacial tissues, the limb bud, and the dorsal root ganglion [9-11].

In this report, we demonstrate that the RCAS-RNAi technique works successfully in the developing chick retina. We show that this loss-of-function method offers distinct advantages. It is long lasting, because the viral vector integrates stably into the genome of infected retinal cells. It is also transmissible, because infected retinal cells release more virus that spread to neighboring cells. Finally, it is easily traced, because each infected cell is clearly marked by viral antigens. This technique thus leads to large patches of infected retinal tissue with knock down of a targeted gene.

## Results and discussion

### RNA interference occurs in chick retinal cells *in vitro*

**Figure 1:**
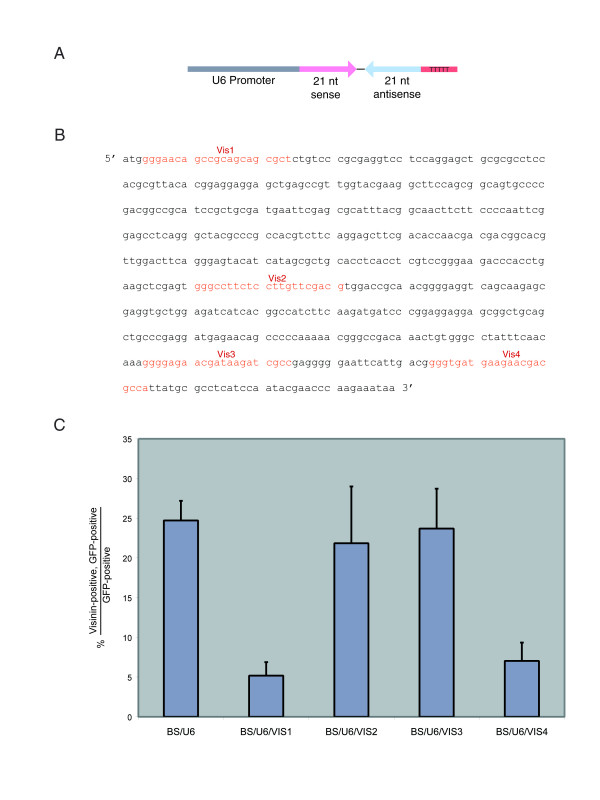
In vitro test of U6-Visinin vector activity on Visinin protein expression (A) A diagram of the BS/U6 vector, showing the murine RNA Polymerase III-specific U6 promoter, the location for oligonucleotide cloning which code for the hairpin, and the 5 consecutive thymidine bases which serve as a termination sequence for RNA Polymerase III. (B) Portions of the visinin sequence used to make hairpins.  Four sequences depicted in red were chosen from the coding sequence.  Each sequence started with three guanine bases, and each sequence was 21 nucleotides long.  Oligonucleotides coding for these sequences were cloned into the BS/U6 vector as described in the Materials and Methods. (C) Electroporation of chick retinas with BS/U6-Vis constructs in vitro.  E6 retinas were electroporated with each construct and a GFP construct, and then were cultured for two days.  Retinas were then dissociated and GFP positive cells were scored for Visinin expression.

The first goal was to establish that RNAi can work in chick retinal cells. A DNA-based RNAi strategy previously shown to produce small hairpins (sh) effective in mammalian cells was used (Figure 1A) [6]. The mouse U6 promoter uses RNA polymerase III to transcribe shRNAs, which are then processed into intermediates that knock down a targeted gene. An RNA polymerase III promoter is used because RNA polymerase III efficiently transcribes short transcripts and then terminates efficiently. A major question was whether the mouse U6 promoter would be compatible with the transcriptional machinery in the chick retina.

Visinin [GenBank: M84729] was chosen as the test gene for RNAi function in the chick retina. Visinin, a calcium-binding protein expressed in photoreceptors, has distinct advantages as a test gene [3]. The visinin transcript is abundant and there are anti-Visinin monoclonal and polyclonal antibodies. Furthermore, visinin reduction should not lead to drastic phenotypes that could confound an assessment of the technique. This prediction is based on the observation that mouse knock outs of recoverin, a mammalian calcium binding protein in photoreceptor cells, only leads to subtle defects in phototransduction [12]. Hairpins were designed against four different regions of the visinin coding sequence. Oligonucleotides coding for the hairpins were then cloned into the pBS/U6 vector.

The shRNA plasmids were co-electroporated with a GFP-expressing plasmid into the explants of embryonic day 6 (E6) retinas. The retinas were then cultured for 2 days, dissociated, and assayed for the number of GFP-positive, Visinin-positive cells. In these experiments, GFP was scored by intrinsic GFP fluorescence and Visinin was detected with a monoclonal antibody (Figure 1C). Constructs with hairpin 2 or hairpin 3 produced the same number of GFP-positive, Visinin-positive cells as control hairpin transfected retina, while hairpins 1 and 4 significantly reduced the number of double-positive cells. These results suggest that hairpin 1 and 4 can reduce the amount of Visinin protein.

### RCAS viruses can deliver RNAi into the chick retina *in vivo*

Electroporation leads only to transient association of DNA with transduced cells and only in limited areas. For a more stable effect that can cover a wide area, the RCAS vector was used to deliver the U6-hairpin. RCAS virus would allow infected cells to continually express the hairpin, because the virus integrates into the cellular genome. Furthermore, these viruses are replication-competent and spread throughout the retina, resulting in patches of infected tissue or a completely infected retina [13-15].

There were two potential complications with an RCAS-U6 hairpin virus. One possible problem is that it contains two promoters, a 5' LTR recognized by RNA polymerase II, which is essential for proper viral replication, and the murine U6 promoter for transcription of the hairpins. It was unclear whether these two promoters would interfere with one another. The second potential problem was that the viral genome and sub-genomic spliced mRNAs might be targeted for degradation, thus reducing viral titers and possibly leading to selection of genomes with the hairpin or U6 promoter deleted.

**Figure 2:**
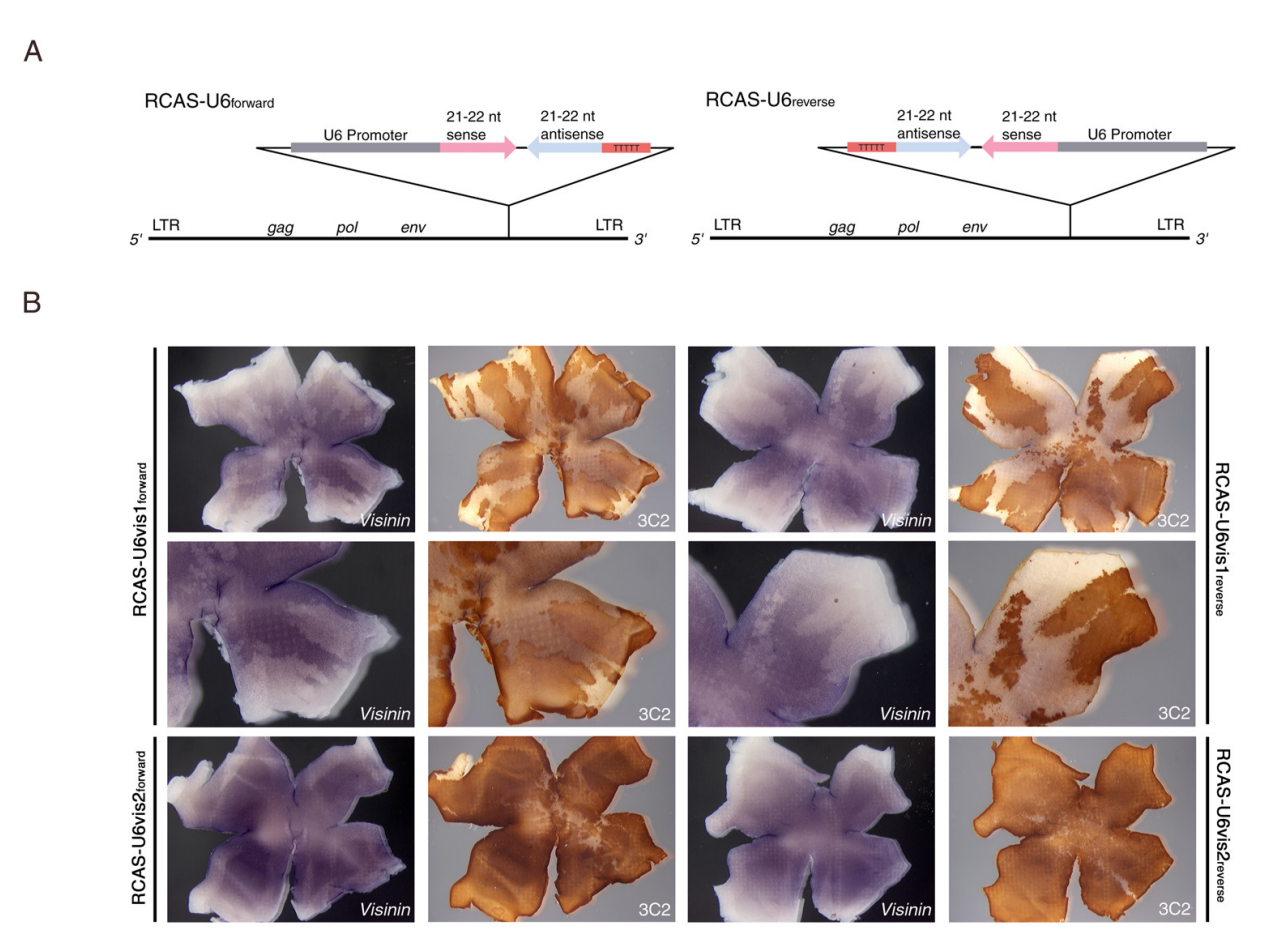
**In vivo test of RCAS-RNAi activity on visinin transcript expression**. (A) A diagram of the RCAS-RNAi vectors tested.  The hairpins were cloned in the forward and reverse directions in order to assess whether orientation altered virus knock down capabilities.  (B) Infection of chick retinas with RCAS-U6-Vis1 and RCAS-U6-Vis2 in vivo.  Stage 10 (~E1.5) retinas were injected with virus and harvested at E6.  Retinas were hybridized with anti-visinin probe and then stained with 3C2 (anti-gag) antibody to allow visualization of virus-infected regions.

To determine empirically if there was an optimum arrangement of the U6 promoter and the hairpin sequence orientation with respect to the LTR and the viral genome, two RCAS constructs were made. One placed the 5'LTR and U6 promoter in the same orientation. The second arranged the two promoters such that they directed transcription in opposite directions. Four different viruses were made using this strategy and hairpins 1 and 2: RCAS-U6-Vis1forward, RCAS-U6-Vis1reverse, RCAS-U6-Vis2forward, and RCAS-U6-Vis2reverse (Figure 2A). All 4 viruses grew to normal titers (~10^8 ^colony forming units/ml), indicating that the viral genome was not successfully targeted by the hairpins that they encode.

When the viruses were introduced into the retina, both RCAS-U6-Vis2 viruses did not reduce visinin expression (Figure 2B). Infected whole mount retinas showed normal visinin expression throughout the retina, with lighter staining in a center spot and equatorial stripe. These results agree with the culture results, in which hairpin 2 did not reduce the number of Visinin-positive cells. Also in agreement with the culture results, both RCAS-U6-Vis1 viruses reduced visinin expression in areas of infection. The knock down was significant, though not complete, perhaps reflecting the efficiency of RNAi. Furthermore, knock down occurred equally with the forward and reverse viruses, suggesting that promoter orientation does not affect viral RNAi activity. The area of knock down also could be manipulated by diluting the amount of virus injected (data not shown).

**Figure 3:**
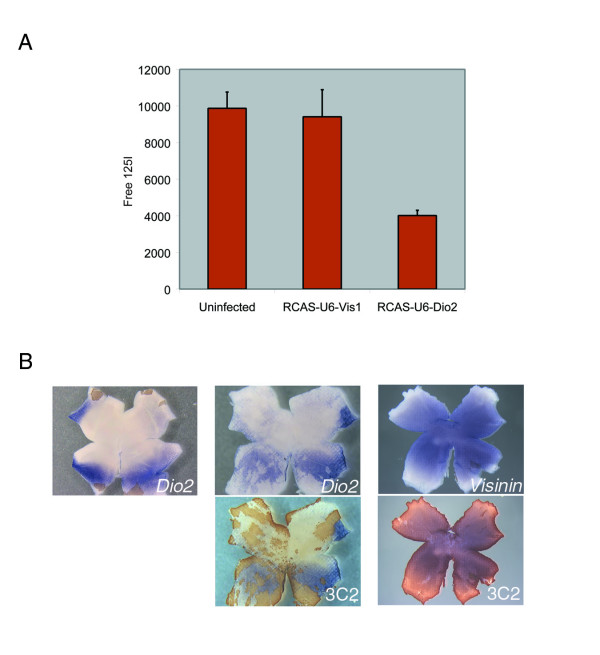
Test of RCAS-RNAi activity on Dio2 protein activity and transcript expression (A) Infection of chick retina with RCAS-U6-Dio2 and biochemical analysis of Dio2 activity.  Stage 10 retinas were injected with RCAS-U6-Vis1, RCAS-U6-Dio2, or left uninfected, and RPE tissue was harvested at approximately E6.  Dio2 activity in the RPE was assayed by measuring free I125 production. (B) Infection of chick retina with RCAS-U6-Dio2 and analysis of Dio2 and visinin expression.  Stage 10 retinas were injected with RCAS-U6-Dio2 and harvested at approximately E6.  Dio2 and visinin transcript expression were assayed by in situ hybridization, followed by viral antigen detection using 3C2 (anti-gag) antibody.

To test whether the RCAS-RNAi vectors can be used more generally in the chick retina, other genes were tested, including the thyroid hormone processing enzyme deiodinase 2 (Dio2) [GenBank: NM_204114] [16]. RCAS-U6-Dio2 viruses grew to normal titers, again suggesting that the RNAi viruses do not target themselves. Dio2 activity was examined in infected eyes using an enzymatic assay. The retinal pigmented epithelium (RPE) was assayed, as this tissue has high Dio2 activity (S. Harpavat and C. Cepko, unpublished observations). RPE infected with RCAS-U6-Vis1 exhibited strong Dio2 activity, similar to uninfected tissue, demonstrating that the RCAS hairpin does not non-specifically affect Dio2. In contrast, RPE infected with RCAS-U6-Dio2 had significantly lower levels of Dio2 activity (Figure 3A). The fact that RCAS-U6-Dio2 infected RPE had some Dio2 activity may reflect the difference between knock down and knock out, or may indicate that the RPE was not fully infected. In addition, reduction of Dio2 transcript in the retina was apparent by in situ hybridization (Figure 3B). The reduction correlated precisely with viral infection, and was specific to Dio2 because RCAS-U6-Dio2 did not change visinin expression. The results demonstrate that in situ hybridization is a convenient way to measure RCAS-RNAi effectiveness.

## Conclusion

We present a technique for gene knock down in the chick retina in vitro and in vivo, using RCAS retroviruses to deliver hairpins mediating RNAi. The viruses were modified with the Gateway sequences (Invitrogen) to facilitate construction. The viruses reduce both transcript and protein levels specific to their targeted gene. Furthermore, because RCAS viruses integrate into the genome of infected cells as well as spread to neighboring dividing cells, RCAS-RNAi infection achieves knock down days after infection in large patches of tissue. Presently we are using the technique to study gene function of a variety of candidates in the developing retina. Furthermore, as we and others have shown, RCAS-RNAi should be useful for loss-of-function studies in any chick tissues accessible to retroviral infection [9-11].

These data indicate that machinery mediating RNAi is present in the chick retina, and that the murine U6 promoter is effective in the chick retina. Furthermore, the hairpins do not induce cell death, as evidenced by 1) the high viral titers obtained in chick fibroblast cells, 2) the large infected patches of retina, and 3) the normal TUNEL staining of infected tissue (data not shown). Interestingly, because RCAS-RNAi viruses grow to normal titers, the viral genome appears protected from the hairpin that it encodes. This may be due to a secondary structure that might form within the viral genome, as well as within the subgenomic spliced mRNA that encodes the env protein. If the target sequence is within a stable secondary structure, it may be inaccessible to the RNAi effects of any transcribed hairpins.

An important caveat is that we have been unable to detect a reduction in RNA levels by in situ hybridization on cryosectioned infected tissue. Cryosectioned tissue shows infection throughout the retinal thickness, in both photoreceptor and non-photoreceptor cells. However, unlike whole mount in situ hybridization which easily detects reduced levels of the target transcript in infected patches, section in situ hybridization shows no detectable difference between infected and uninfected areas. It is still unclear why this is the case, and may be related in part to the thickness of retinal sections. Perhaps if RNAi led to complete absence, instead of knock down, section in situ hybridization would be a better method of screening for functional RCAS-RNAi constructs.

## Methods

### Cloning of RNAi constructs

The RCAS-RNAi viruses have been modified so that they are now compatible with the Gateway cloning system (Invitrogen). DNA encoding the hairpin is cloned into a modified U6 vector using standard cloning techniques (see additional information PDF for detailed protocol). The construct is then moved into the retroviral vector using Gateway reagents, thus bypassing the difficulties of cloning small fragments into RCAS. As a result, each construct can be made with a minimal number of cloning steps. See Supplemental Information for a detailed protocol.

Before the Gateway modification, hairpins were cloned into the pBS/U6 vector as previously described [6]. Briefly, single-stranded oligonucleotides corresponding to the 5' and 3' ends of the hairpin were annealed together. The 5' fragment was engineered with a 5' Apa I site and 3' HindIII site, whereas the 3' fragment was engineered with a 5' HindIII site and a 3' EcoRI site. Both fragments were cloned into an ApaI-EcoRI cut pBS/U6 vector in a triple-ligation reaction. Inserts were deemed correct if they produced a ~465 bp insert with T3–T7 PCR. The resulting BS/U6-hairpin fragment was isolated by KpnI and EcoRI digestion, blunted, and cloned into the ClaI site (blunted) of RCAS. Inserts in both the forward and reverse direction were identified by XbaI digestion.

### Immunohistochemical detection of knock down

pBS/U6 or pBS/U6-hairpin vectors were electroporated into dissected E6 chick retina using an in vitro electroporation approach. First, retinas were dissected in prewarmed Hanks' Balanced Salt Solution 1X (Cellgro) and their RPE removed. They were then placed in a bath containing ~200 ng/ul BS/U6-hairpin plasmid DNA and ~200 ng/ul CAG-GFP plasmid in 1XPBS, and exposed to 5 pulses of 25 volts, 50 milliseconds each. Retinas were then removed from the electroporation chamber and placed on Nuclepore Track-Etch membranes (Whatman, 25 mm diameter, 0.2 um pore size) floating atop culture media (50% F12 Nutrient Mix (Gibco), 40% Dulbecco's Modified Eagel's Medium (Gibco), 10% Fetal Calf Serum (HyClone), 2 mM L-glutamine (Gibco), Penicillin/Streptomycin (Gibco), and 5 ug/ml insulin (Sigma)). After two days in culture at 37 degrees Celcius and 5% CO2, retinas were dissociated and stained as previously described [17]. Retina were dissociated in a light trypsin solution, and cells were plated on poly-D-lysine coated slides. Cells were blocked with a 10% goat serum, 0.1% Tween, 1XPBS solution, and stained with anti-visinin monoclonal antibody GH9 (1:500) and goat anti-mouse Cy3 secondary antibody (1:200) in block. Cells were also treated with DAPI to identify all cell nuclei.

Cell scoring was performed blindly by the same observer. This was done to ensure that cells were scored consistently as "positive" or "negative" among the different samples. Many cells were clearly positively or negatively stained; for those cells that were stained less clearly, the observer used the same criteria to determine a cell's designation.

### Viral injection

RCAS-RNAi viruses were made by transfecting DF-1 cells with viral DNA. Cells were grown for 14 days, after which the virus was harvested, concentrated, and titered as described previously [15]. Stage 10 chick embryos were injected with virus in the optic vesicle, and embryos were allowed to grow until E6–7. Retinas were harvested from injected embryos, fixed, and dehydrated in methanol in preparation for in situ hybridization. In these experiments, viral injection as opposed to in ovo RCAS-RNAi DNA electroporation was chosen because viral injection leads to more uniform areas of infection and less chances of tissue damage from electroporation. However, in ovo RCAS-RNAi DNA electroporation can also be used successfully in the chick retina (data not shown).

### Whole mount in situ hybridization detection of knock down

Whole mount in situ hybridization was performed as previously described [18]. Retinas were dissected, fixed in 4% paraformaldehyde, and dehydrated in increasing concentrations of methanol. Retinas were then rehydrated, cut, and placed between two mesh pieces in order to keep them flat. Meshed retina were treated with Proteinase K, fixed with paraformaldehyde and glutaraldehyde, placed in prehybridization solution, and then exposed to probe overnight at 70 degrees. The following day meshed retina were washed in formamide and detergents extensively at 65 degrees, followed by a block and anti-DIG antibody incubation overnight. The third day consisted of many hour-long washes in normal salt solution, and the final day consisted of detection using a combination of NBT and BCIP to produce a purple precipitate. Antisense probes made against the entire coding sequences of Visinin and Dio2 were used. After taking pictures of the in situ hybridization result, retinas were then stained with anti-gag monoclonal antibody AMV-3C2 (DSHB, University of Iowa) to identify areas of viral infection [19].

### Deiodinase assays

Dio2 assays were performed using standard methods [16]. 40 ug of protein were incubated per sample in two different reactions with 125I-T4. The first reaction used 1 nM T4, a concentration too low to inhibit Dio2 activity. The second reaction used 1 uM T4 which inhibits Dio2 activity. Samples were incubated for 3 hours at 37 degrees, and the amount of free 125I was then measured as an indication of Dio2 activity. The difference between the 1 nM T4 and 1 uM T4 experiments represented the amount of Dio2 activity in the tissue.

## Authors' contributions

SH and CC conceived the technique, designed the vectors, conducted the experiments, and wrote the manuscript.

## Supplementary Material

Additional File 1 **Protocol for making an RCAS-RNAi virus**. PDF file listing step-by-step how to create viruses that knock down genes in the chick retina. Click here for file
