# Supplementary material for: RCAS-RNAi: A loss-of-function method for the developing chick retina
Source: BMC Dev Biol. 2006 Jan 22;6:2. doi: 10.1186/1471-213X-6-2 (PMC1402266; doi:10.1186/1471-213X-6-2)
Supplement: Additional File 1 — Protocol for making an RCAS-RNAi virus. PDF file listing step-by-step how to create viruses that knock down genes in the chick retina. [file 1471-213X-6-2-S1.doc]

**RCAS-RNAi: A loss-of-function method for the developing chick retina: Cloning Protocol**

**This protocol is designed to hasten cloning small fragments into RCAS. It involves six steps that can be completed over the course of a week:**

1. **Preparing the pCS6/U6 vector**
2. **Annealing the oligonucleotides**
3. **Performing the triple ligation**
4. **Screening ligations results**
5. **Performing the BP/LR Gateway reactions**
6. **Screening Gateway results**

**Step 1: Preparing the pCS6/U6 vector ("pCMV-Sport6/RNA Pol III U6 promoter" vector)**

The pCS6/U6 vector has two advantages: (1) Gateway compatibility, and (2) ampicillin resistance. Because of Gateway, this vector goes through a ligation reaction only once; all the other cloning steps are performed through the very efficient Gateway technology. With ampicillin selection, this vector does not need a one-hour recovery time following transformation.

The pCS6/U6vector was made by ligating an M13 PCR product from the pBS/U6 vector (from the Shi lab, Harvard Medical School) to a Rsr II-Mlu I blunted pCMV-Sport6 vector. The map of the vector is as follows:


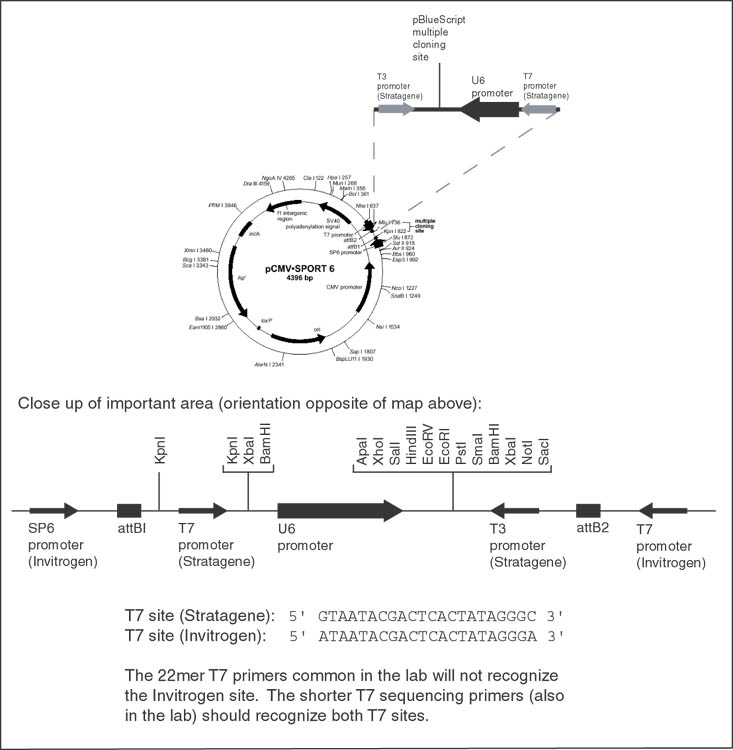


To prepare the vector, first perform an Apa I digest, then a T4 polymerase fill in, then an EcoRI digest, and then a CIP reaction:

|  | 1. ApaI digest  20 ul miniprep  10 ul NEB#4  10 ul BSA 1mg/ml  5 ul Apa I  55 ul water  100  Let reaction run ~2 hr RT,  Then purify using PCR purification kit and elute in 40 ul water |  | 2. T4 Polymerase Fill In  37.5 ul PCR purified product  10 ul 5X T4 Polymerase buffer  2 ul T4 Polymerase  1 ul 10mM dNTP  50  Let reaction run 30 minutes, 37 degrees  Then purify using PCR purification kit and elute in 88 ul water |
| --- | --- | --- | --- |
|  |  |  |  |
|  | 3. EcoRI digest  85 ul PCR purified product  10 ul Buffer H  5 ul EcoRI  100  Let reaction run ~2hr, 37 degrees  Then purify using PCR purification kit and elute in 30 ul water |  | 4. CIP reaction  30 ul PCR-purified product  3.5 ul CIP 10X buffer  1.5 ul CIP  35 ul  Let reaction run 30 minutes, 37 degrees  Then gel purify product (expect ~4600 bp piece and small piece – cut ~4600 bp piece) |

(Vector is CIP'd in this case is to minimize self-ligation without insert)

**Step 2: Annealing the oligonucleotides**

First, pick the appropriate gene sequences that will comprise the hairpin. A common strategy with the U6 promoter system is to look for sequences starting with 3 guanine nucleoides in sequence. Various other rules have been suggested in order to pick sequences that make the best hairpins, with a higher GC count (above 45%) thought to be better. However, for the chick retina no reproducible rule for picking effective hairpins has been found. It is recommended making multiple hairpins using sequences from different parts of the targeted gene and testing the constructs empirically.

This technique requires ordering 4 oligonucleotides for a given 21 nucleotide sequence that fit together as follows:


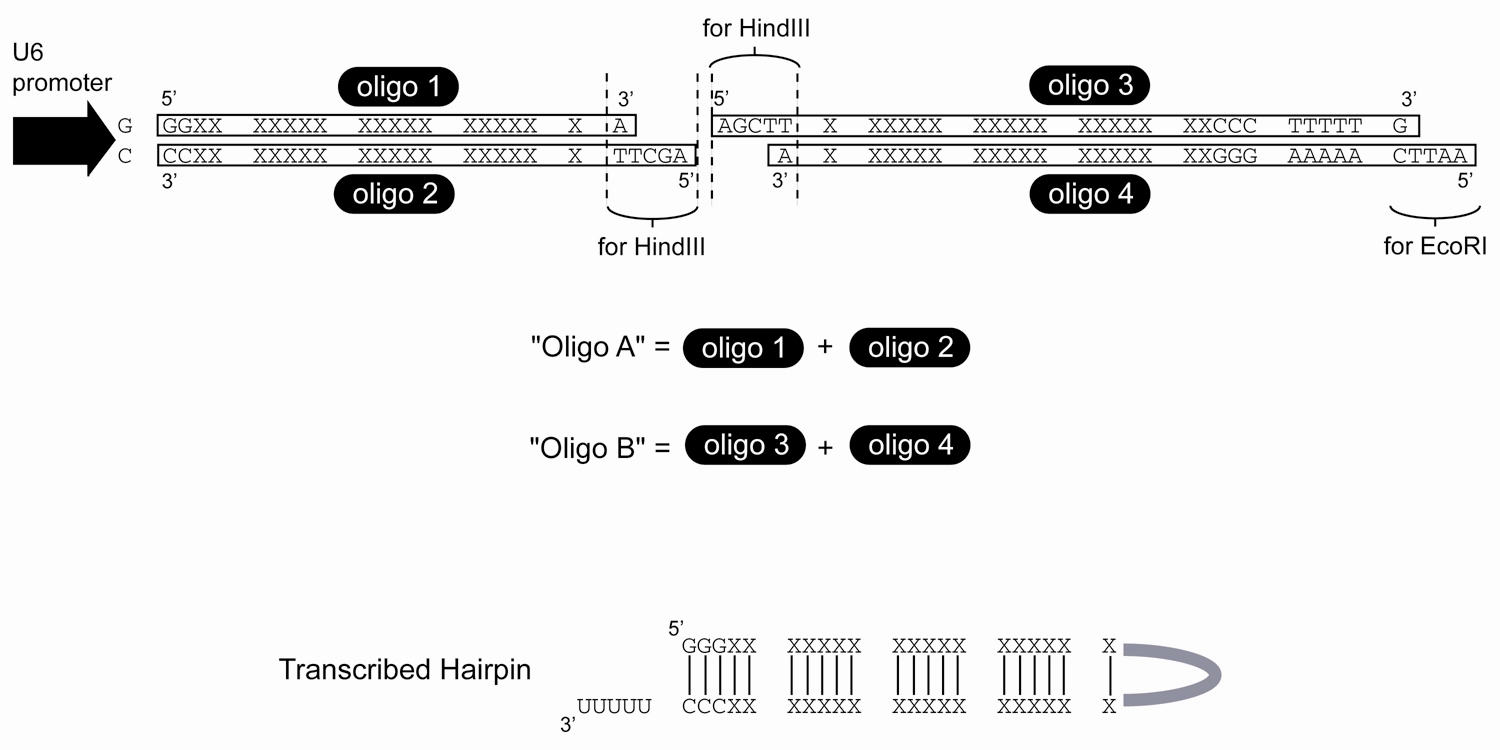


Once the sequences have been chosen, they have to be annealed. This requires phosphorylating them so that they can ligate together in the triple ligation. It also involves purifying the oligonucleotides. Unfortunately, the oligonucleotides are too small to be retrieved with the PCR purification columns, so a phenol/chloroform step is needed. The fastest way to do all of this is through technique adapted from Takahiko Matsuda, Cepko Lab, Harvard Medical School (perform same reaction for oligo 3 and oligo 4):

|  | 1. Annealing   5 ul 10X buffer H  6 ul 100uM oligo 1  6 ul 100uM oligo 2  33 ul water  50 ul  Boil 800ml water in 1L beaker, place tubes in beaker for 1 minute, and then remove beaker (with tubes inside) from heat plate and let cool on bench for 30 minutes |  | 1. Phosphorylate Annealed Oligos   2 ul Annealed Oligo  1 ul 10x PNK buffer  1 ul 10mM ATP  1 ul PNK  5 ul water  10 ul  Let reaction run 30 minutes, 37 degrees |
| --- | --- | --- | --- |
|  |  |  |  |
|  | 1. Purification   Add 90 ul water/PNK reaction  Add 100 ul Phenol/chloroform  Vortex quickly  Spin at maximum speed 3 minutes  Recover upper phase |  | 1. Precipitation   100 ul upper phase  300 ul 100% Ethanol  10 ul 3M NaOAc  1 ul Glycogen Carrier  Spin 13K, 5 minutes  Remove supernatent  Add 500 ul 70% EtOH  Spin 13K, 2 minutes  Resuspend in 100 ul water |

**Step 3: Performing the triple ligation**

Because the Takara I ligation reagent (http://takaramirusbio.com) is very efficient, it is preferred for the ligation steps:

|  | Experimental ligation:  1 ul vector  1 ul oligo A  1 ul oligo B  3 ul Takara I reagent  6 ul |  | Control ligation:  1ul vector  2 ul water  3 ul Takara I reagent  6 ul |
| --- | --- | --- | --- |

This ligation can be performed at 16 degrees overnight, or it can be accomplished at room temperature for 30 minutes. Following the ligation, the DNA can be transformed by adding 6 ul ligation mix to 50 ul DH5a cells, heat shocking for 1.5 minutes at 42 degrees, recovering on ice for 2 minutes, adding 56 ul LB broth, and plating the entire 112 ul on AMP plates (recovery for 1 hour is not needed). Expect to see some colonies on the control plates, but many more on the vector+oligo plates.

**Step 4: Screening ligation results**

Many colonies that grow should be correct, but some may contain multiple inserts or the wrong sequence. To test these possibilities, there are many options including colony PCR, Bam HI digestion, and sequencing.

Colony PCR is nice because it saves time (no need for cultures to grow overnight) and money (no need to do extra minipreps). Pick individual colonies, suspend each in 30 ul 1XPCR buffer, and perform PCR as follows:

|  | PCR mix/rxn:  1 ul 10XPCR buffer  1 ul 10uM T7 long primer  1 ul 10uM T3 primer  0.5 ul 10mM dNTPs  0.2 ul Taq  5.3 ul water  10 ul bug in 1XPCR buffer  20 ul |  | PCR program:  1. 92 degrees, 1 minute   1. 92 degrees, 30 seconds 2. 58 degrees, 30 seconds 3. 72 degrees, 1 minute 4. Go to 2, 30X 5. 72 degrees, 5 minutes 6. 4 degrees, infinity |
| --- | --- | --- | --- |

As a control, use the pCS6/U6 vector DNA. The colonies with the correct inserts should produce a fragment indistinguishable from the pCS6/U6 vector product (remember: the uncut vector has sequence between the Apa I and EcoRI sites, which are approximately the same length as the inserted hairpin). 1.5-2% gels work best for this analysis, using marker VIII or V. For those colonies with the correct size, proceed with overnight cultures and minipreps. For overnight cultures, add 5 ul of colony in 1XPCR buffer to 3.5 ml LB broth + ampicillin and shake at 37 degrees.

Digesting with Bam HI is another way to test the colonies, although it first requires miniprepping. One approach is the following:

|  | BamHI digest:  2 ul miniprep DNA  3 ul 10X Buffer B  2 ul BamHI  23 ul water  30 ul |  |
| --- | --- | --- |

Again, for a control use pCS6/U6 vector. The correct inserts produce an ~400bp fragment indistinguishable from the control digest product.

Finally, sequencing is an ideal way to confirm your product. However, many sequencing protocols cannot efficiently sequence through hairpins. One option is to first cut with HindIII and then send the sample for SP6 and T3 sequencing.

**Step 5: Performing the BP/LR Gateway reactions**

The Gateway reactions are really straightforward, and are best explained in the Gateway catalog at the Invitrogen website (www.invitrogen.com). Consult this manual to learn how to deal with the temperature sensitive clonase enzymes. The strategy involves cutting the pCS6/U6+insert vector with enzymes in the backbone to ensure that the vector will never re-ligate on itself. Then the vector is run through the BP and LR Gateway reactions (the reason why the vector goes through both reactions in this protocol is to completely avoid relying on the more tedious kanomycin resistance selection).

| 1. NcoI/ScaI vector backbone digest:  10 ul pCS6/U6+insert  3 ul 10X Buffer H  1.5 ul Nco I  1.5 ul Sca I  14 ul water  30 ul  Let reaction go 37 degrees overnight to ensure complete cutting, then purify using PCR purification kit and elute in 30 ul water  Note: make sure NcoI and ScaI do not cut in the insert! |  | 2. BP reaction:  4 ul BP reaction buffer  8 ul NcoI-ScaI cut DNA  2 ul pDONOR 201 150ng/ul  2 ul TE ph 8.0  4 ul BP Clonase Mix  20  Consult catalog for instructions on how to use Clonase Mix enzymes (they are temperature sensitive).  Let reaction run at RT for 3-4 hours |  | 3. LR reaction:  20 ul original tube  1 ul 0.75 M NaCl  3 ul pG-RCAS(A)  6 ul LR Clonase Mix  30 ul  Consult catalog for instructions on how to use Clonase enzymes (they are temperature sensitive).  Let reaction sit at RT for 1-2 hours, then add 3 ul Proteinase K for 10 minutes, 37 degrees  The pG-RCAS vector is an RCAS vector that is gateway compatible. |
| --- | --- | --- | --- | --- |

Following the Gateway reactions, transform 1ul of the reaction mix into 50 ul DH5a cells. As a negative control, transform 1 ul of the NcoI-ScaI digest into 50 ul DH5a cells. The negative control should not give colonies. If many colonies do appear in the negative plates, it means that the original NcoI/ScaI vector digestion was incomplete.

**Step 6: Screening Gateway results**

Pick 2-4 colonies (the Gateway reaction is very efficient, and most colonies should be correct) and set up 3 ml ampicillin cultures overnight. Miniprep 1.5 ml the next day (save the other 1.5 ml for maxiprepping), and perform the following digestion:

|  | Xba I digestion:  4 ul DNA  3 ul Buffer H  2 ul Xba I  21 ul water  30 ul  Let reaction run at 37 degrees for 2 hours |  |
| --- | --- | --- |

From the Xba I digest, correct constructs have 4 bands: ~7872 bp, ~2303 bp, ~1347 bp, and ~400 bp. Take 5 ul of the correct culture (you should have 1.5 ml left) and add to 100 ml LB + ampicillin for overnight maxiprep culture.
